# Supplementary material for: Content-rich biological network constructed by mining PubMed abstracts
Source: BMC Bioinformatics. 2004 Oct 8;5:147. doi: 10.1186/1471-2105-5-147 (PMC528731; doi:10.1186/1471-2105-5-147)
Supplement: Additional File 5 — The original Chilibot query results of the term "long-term potentiation (LTP)" and 22 other terms, limiting the latest references analyzed to the years 1990, 1995, 2000, and 2004. [file 1471-2105-5-147-S5.bz2 › chilibotAdditionalFile5/ltp1995/html/NMDA_ACTIN.html]

 


 **NMDA** and **ACTIN** 
  
Found 6 abstracts in PubMed,  **6 abstracts were retrieved and analyzed**.  


---

 Search Google  |
 PDF files only 
|  EDU domain only 

---

**Interactive relationship** (e.g. stimulation, inhibition, etc)

- Calcium induced  **actin**  depolymerization reduces  **NMDA**  channel activity.  Ref: 7684233 Neuron, 1993
- Our results suggest that calcium and ATP can influence  **NMDA**  channel activity by altering the state of  **actin**  polymerization and are consistent with a proposed model in which  **actin**  filaments compartmentalize a channel regulatory protein.  Ref: 7684233 Neuron, 1993

**Parallel relationship** (e.g. studied together, co-existance, homology, etc.)

- We tested whether  **actin**  depolymerization influences  **NMDA**  channel activity in whole cell recording on cultured hippocampal neurons.  Ref: 7684233 Neuron, 1993
- The identification of p87 as the  **actin**  binding MARCKS protein and the demonstration of its presence in neurites and growth cones of granule cells suggests that it may be involved in  **NMDA**  stimulated neurite outgrowth.  Ref: 8261132 Eur J Neurosci, 1993
- The ATP and calcium dependent rundown of  **NMDA**  channels was prevented when  **actin**  depolymerization was blocked by phalloidin.  Ref: 7684233 Neuron, 1993
- When the presence of  **actin**  rich structures was examined using rhodamine phalloidin labelling it was found that  **NMDA**  increased the proportion of neuronal processes that possessed a growth cone by 28%.  Ref: 1355016 Brain Res Dev Brain Res, 1992
- Cytochalasins, which enhance  **actin**  ATP hydrolysis, induced  **NMDA**  channel rundown.  Ref: 7684233 Neuron, 1993
